# The modulatory effects of autonomous sensory meridian response (ASMR) on pain

**DOI:** 10.1097/PR9.0000000000001487

**Published:** 2026-08-05

**Authors:** Agnieszka Janik McErlean, Joseph Walsh, Katie Nash, Paige-Shannon Harper

**Affiliations:** a School of Sciences, Bath Spa University, Bath, United Kingdom; b University of the West of England, Bristol, United Kingdom

**Keywords:** ASMR, Autonomous sensory meridian response, Pain, Pain appraisal, Pain threshold, Sex differences, Pain processing

## Abstract

**Introduction:**

Autonomous sensory meridian response (ASMR) is a cross-sensory phenomenon experienced by an estimated 20% of the population and characterised by a calming, pleasurable tingling sensation triggered by audio–visual or tactile stimuli. Prior work suggests that ASMR experiencers show heightened pain sensitivity but similar pain tolerance to nonexperiencers, and emerging evidence points to possible analgesic effects.

**Objectives:**

This study sought to replicate and extend these findings using a larger sample, pain threshold as an index of pain perception, and sex as a variable.

**Methods:**

Male and female ASMR experiencers (N = 20) and controls (N = 20) completed algometer testing at baseline, during an ASMR-inducing video, and during a control video. Pain thresholds and visual analogue pain ratings were collected, and participants reported whether they experienced ASMR tingling in each condition.

**Results:**

ASMR experiencers reported higher pain ratings than controls (*P* = 0.017) but showed comparable pain thresholds (*P* = 0.963). Among ASMR experiencers, experiencing tingles was associated with increased pain threshold (*P* = 0.012). Women overall exhibited lower thresholds (*P* = 0.001) and showed no condition-related changes, whereas men demonstrated increased thresholds during both videos (*P* = 0.009, *P* = 0.007), suggesting a distraction-related buffering effect for men. Descriptively, male ASMR experiencers mirrored this pattern, while female ASMR experiencers showed elevated thresholds only during the ASMR video.

**Conclusion:**

These findings indicate that ASMR may modulate pain perception and that sex differences influence pain-related responses. They highlight ASMR's potential as an analgesic mechanism and underscore the importance of incorporating sex as a factor in future ASMR pain research.

## Abstract 2

Supplemental Digital Content is Available in the Text.

Autonomous sensory meridian response (ASMR) increases pain thresholds in experiencers pointing to its analgesic properties. However, different mechanisms are proposed to drive ASMR's pain-modulating effects for men and women.

## 1. Introduction

Autonomous sensory meridian response (ASMR) is a multisensory phenomenon, which entails experiencing a scalp-centred tingling sensation which can radiate throughout the body leading to a relaxed and calm state.^3,19^ Recent estimates place the prevalence of ASMR at 20% in the general population suggesting this to be a common phenomenon.^29^ Autonomous sensory meridian response can be triggered by stimuli, such as whispering, personal attention, and touch, in day-to-day life and through intentionally created videos and audio recordings containing these triggers.^3,19^ Individuals who engage with ASMR inducing media do so to elicit the pleasurable sensation and to promote relaxation and sleep.^3,19^ Autonomous sensory meridian response has also been linked to elevated mood and reduced heart rate, suggesting the phenomenon has a distinct emotional and physiological component.^8,17,26^

Barratt and Davis^3^ proposed that ASMR can have analgesic properties as 42% of their sample of ASMR experiencers who suffered from chronic pain reported a reduction in pain symptoms for several hours after experiencing the sensation. In our own previous study,^21^ we found that compared with controls, ASMR experiencers reported elevated subjective pain appraisal, indexed by visual analogue scale ratings in response to the application of an algometer. No group differences were found in terms of the pain levels measured with tolerance scores. Increased sensitivity to exteroceptive nociceptive tactile stimulation found among ASMR experiencers in our study is intriguing and broadly consistent with neuroimaging findings of heightened activity within brain areas linked with somatosensation and pain processing,^25,33^ and self-reports of increased sensory sensitivity among ASMR experiencers compared to controls.^20,27,31,33^ In addition, we found that descriptively the highest pain tolerance and the lowest pain ratings were observed among ASMR experiencers when they were asked to watch an ASMR video compared with a control video and at baseline, pointing to a possibility of the analgesic properties of experiencing ASMR,^21^ which is broadly consistent with Barrat and Davis's findings.^3^ Interestingly, increased parasympathetic activation, previously linked with lower pain intensity^13,35^ and increased pain tolerance,^9,12,30^ has also been found to underpin ASMR.^8,17,19^ However, a simultaneous activation of parasympathetic and sympathetic branches in ASMR has also been reported.^26^

The aim of this study was to replicate previous results with a larger sample and to include sex as a variable, given established sex-based differences in pain perception, reporting, and coping,^11,23,37^ to determine whether ASMR differentially modulates pain experience in men and women. A secondary aim was to establish if previous findings extend to pain threshold in response to the application of an algometer as a related but different pain measurement. Last, we wanted to establish whether ASMR experiencers would show reduced pain levels in the ASMR video condition compared with other conditions, which would further indicate ASMR's potential to alleviate pain.

## 2. Method

### 2.1. Participants

A priori power analysis using gpower^10^ for the 3 planned 2 (sex: male, female) × 2 (group: ASMR experiencers; controls) × 3 (video type: ASMR, control, no video) mixed ANOVAs to obtain α = 0.05, power (1 − β) = 0.80, medium effect size = 0.25, indicated a sample size of 40. Sixty-three participants were recruited among university students and a snowballing technique. All participants in the final sample were either male or female and identified with the sex they were assigned at birth. Three participants stated they did not identify with the sex assigned at birth and were therefore excluded from the analysis. All participants aged between 18 and 40 years, with no chronic pain diagnosis, and had not taken part in our previous study examining the impact of ASMR on pain experience. In exchange for their time, participants were offered £5 Amazon vouchers or credits as part of the university's research participation scheme.

Participants were classified as ASMR experiencers or controls, based on their responses to a trigger checklist used in previous studies including the study on pain processing in ASMR.^20,22,27^ To be classified as an ASMR experiencer, participants needed to select tingling intensity of minimum 1 to at least 1 common trigger, with most ASMR experiencers selecting multiple triggers and indicating intensity above 0 for several listed stimuli. They also had to indicate that the sensation is pleasurable and calming^34^ (see Appendix, http://links.lww.com/PR9/A437 for full checklist and the verification procedure). Eighteen participants who could not be firmly classified either as controls or ASMR experiencers due to their inconsistent answers on the ASMR questionnaire were excluded from the analysis.

In addition, 2 outliers scoring > 3*SDs* on a number of variables were also removed from the analysis. The final sample size N = 40 comprised 20 ASMR experiencers (F = 11, M = 9, age: *M* = 21.60, *SD* = 3.98) and 20 controls (F = 9, M = 11, age: *M* = 21.35, *SD* = 1.35). Groups did not differ in terms of age *t* (38) = 0.27, *P* = 0.792 *M*_diff_ = 0.25, 95% CI [−0.54, 0.70] or gender [χ^2^ (1, Ν = 40) = 0.40, *P* = 0.527].

### 2.2. Materials

#### 2.2.1. Stimuli

To ensure consistency, we used the same 2 video clips we had previously included in our study on pain perception among ASMR experiencers.^21^ Both were sourced from YouTube and played for 3 minutes each. One video was intentionally created to induce ASMR and featured a visit to a hairdresser and contained several common ASMR triggers including personal attention, slow and deliberate hand movements, and crisp sounds, thus increasing the likelihood of triggering ASMR. The control video featured a tutorial on vegetable peeling and similarly to the ASMR video it was filmed close-up, and while it included trigger-like stimuli such as giving instructions and repetitive hand movements, these were not slow and smooth as in ASMR videos and people in the video spoke in their normal voices rather than whisper or soft voice typically used in ASMR videos. Based on that we expected that the control video would be less likely to result in an ASMR experience.

#### 2.2.2. Pain induction (threshold)

Manual algometer (Wagner Instruments, 1217, Greenwich, CT 06836, United States of America FPX series) was used for the pain induction procedure. This entailed applying pressure with the device to the soft tissue of the dorsal forearm, approximately 8 cm below the elbow joint, via a 1 cm^2^ rubber surface according to guidelines.^7^ Researchers were trained to apply gradually increasing pressure to the induction site, at a rate of approximately 0.5 kg/cm^2^ per second, per the recommendation of previous research.^1,6^ An algometer measures the pressure (ft/lbs) which produces a pressure-pain threshold score and can be applied several times in close succession without carry-over effects making it a desirable experimental procedure.^24^ Algometer pain induction was chosen to align with the authors' previous work^21^ and in recognition of wider research which demonstrates that ASMR responses are strongly associated with tactile sensitivity.^27^

During induction, participants were asked to verbally report their pain threshold which is the point at which they started to experience a painful sensation. To reduce measurement error, this was repeated 3 times in each condition (at the start, after approximately 1 minute and 2 minutes, see Appendix, http://links.lww.com/PR9/A437 for details), and the average of the 3 algometer measurements was calculated to obtain the participant's pain threshold for that condition. To reduce the possibility of fatigue, the algometer was moved slightly each time ensuring that it was always within 8 cm of the elbow joint on the dorsal forearm of the same side. The algometer was always applied to the nondominant arm, in light of research suggesting differences in pain appraisal between dominant and nondominant arms.^36^

##### 2.2.2.1. Pain appraisal

Participants gave subjective pain ratings after each algometer induction session using a visual analogue scale (VAS), which has been widely used in broader research.^4,15^ The VAS used in this study was 10-cm long and ranged between 0 (no pain) to 100 (pain as bad as it could be). Participant's score was determined by measuring the distance (in mm) between 0 and the participant's mark with a ruler with higher scores indicating greater pain intensity. This measure reflects participants' subjective appraisal of their pain experience.

##### 2.2.2.2. Tingle frequency

To determine whether participants experienced the ASMR sensation in the 3 conditions, they were asked to indicate the frequency of the experienced tingles on a scale from 1 (none of the time) to 7 (all of the time) at baseline and after the ASMR and control videos.

### 2.3. Procedure

Once the participants read the Information Sheet and provided consent, they then completed the online ASMR checklist which was subsequently used to classify them as ASMR experiencers or controls. Participants then completed the baseline, ASMR video, and control video conditions in a counterbalanced fashion. In the ASMR video condition, the algometer was administered to coincide with ASMR triggers and in the control video to coincide with ASMR-like triggers (see Appendix, http://links.lww.com/PR9/A437 for details). Participants were asked to verbally indicate when they could first register the painful sensation at which point the pain induction was stopped and the threshold value recorded. The 3 scores were averaged to calculate the pain threshold for each condition. Participants then completed the VAS to indicate their pain appraisal from the administration of the algometer, providing both subjective appraisal scores and pain measurements. They also completed the tingle frequency scale in each condition to indicate whether they experienced ASMR. This study was approved by the Ethics Review Panel at Bath Spa University.

### 2.4. Design and analytical approach

To determine whether there are differences between ASMR experiencers and controls, and between men and women, across baseline (no video), ASMR video, and a control video condition, a repeated measures experimental design was implemented, and three 2 (group: ASMR experiencers, controls) × 2 (sex: male, female) × 3 (video type: ASMR, control, no video) ANOVAs were planned for each of the 3 dependent variables (tingle frequency, pain threshold scores measured with an algometer, and VAS scores).

## 3. Results

Statistical analysis was conducted using JASP.^18^ Assumptions checks output and anonymised data are available online at https://osf.io/5689t/?view_only=d44f79714918483e9ef6b4dbd50d72ba.

### 3.1. Tingle frequency

**Figure 1.**
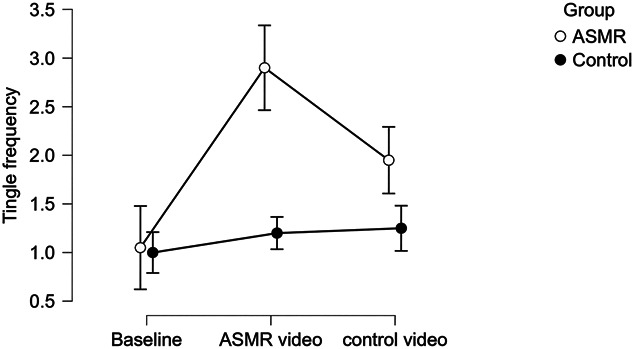
Tingle frequency scores across baseline, ASMR video, and control video conditions for ASMR experiencers and controls. ASMR, autonomous sensory meridian response.

A 2 (group: ASMR experiencers, controls) × 2 (sex: male, female) × 3 (video type: ASMR, control, no video) ANOVA was planned on the tingle frequency scores. However, due to the violations to normality and homogeneity of variance assumptions 3 Welch tests were conducted instead to establish whether the groups differed in terms of the intensity of their tingles across the no video, ASMR video, and control video conditions. This revealed 2 statistically significant results showing that ASMR experiencers (*M* = 2.90, *SD* = 1.48) reported higher tingle frequency than controls (*M* = 1.20, *SD* = 0.52) in the ASMR video condition (*Welch's F* [1, 23.65] = 23.37, *P* = 0.001, *d* = 1.53) and in the control video condition, although the group difference here was not as pronounced (ASMR experiencers: *M* = 1.95 *SD* = 1.19; Controls: *M* = 1.25, *SD* = 0.72, *Welch F* [1, 31.16] = 5.07, *P* = 0.031, *d* = 0.71) (Fig. 1).

**Figure 2.**
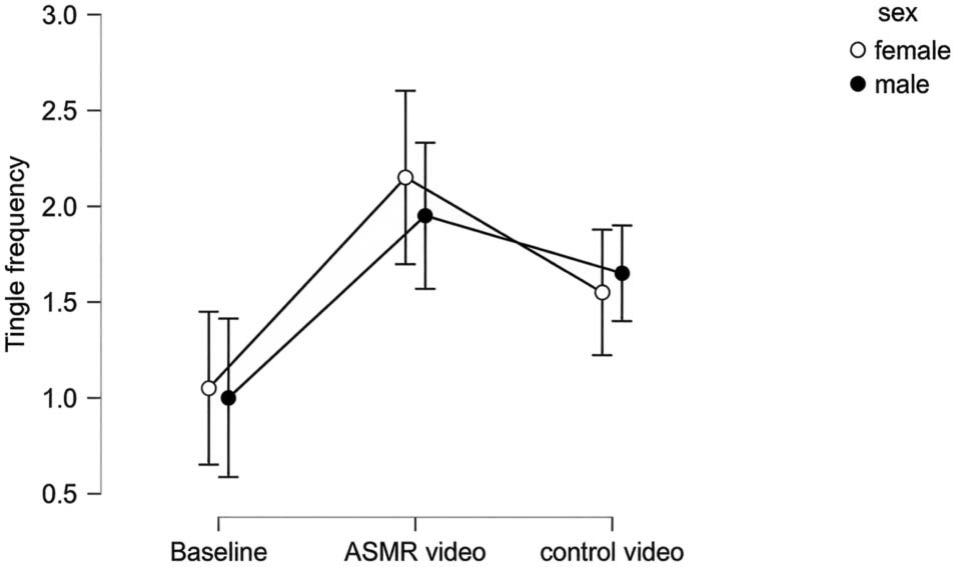
Tingle frequency scores across baseline, ASMR video, and control video conditions for men and women. ASMR, autonomous sensory meridian response.

Another set of 3 Welch tests was conducted to establish if there were sex differences with respect to the 3 conditions, which resulted in no statistically significant results (Fig. 2).

Two separate Friedman tests were also conducted to examine the tingle frequency data within each group, which yielded a nonsignificant result for the control group and statistically significant results for the ASMR experiencers (χ^2^ *F* [2] = 25.15, *P* < 0.001, *Kendall W* = 0.63). This was followed up with Bonferroni-corrected Conover post hoc tests which revealed statistically significant differences between all 3 conditions: (1) baseline vs ASMR video (*t* (38) = 8.00, *r*_*rb*_ = −1.00, *P* = 0.001), (2) baseline vs control video (*t* (38) = 3.54, *r*_*rb*_ = −1.00, *P* = 0.003), and (3) ASMR and control video (*t* (38) = 4.46, *r*_*rb*_ = 0.81, *P* < 0.001) with ASMR experiencers indicating higher frequency of their tingles in the ASMR video condition (*M* = 2.90, *SD* = 1.48) compared with baseline (*M* = 1.05, *SD* = 0.22) and to the control video condition (*M* = 1.95, *SD* = 1.19).

### 3.2. Visual analogue scale pain scores

**Figure 3.**
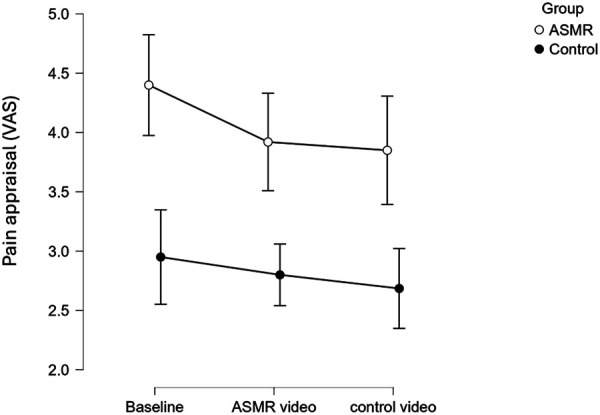
Pain appraisal scores indexed by VAS ratings across baseline, ASMR video, and control video conditions for ASMR experiencers and controls. ASMR, autonomous sensory meridian response; VAS, visual analogue scale.

All assumptions have been met therefore, a 2 (group: ASMR experiencers, controls) × 2(sex: male, female) × 3 (video type: ASMR, control, no video) ANOVA was performed on the subjective pain level experienced in each condition indexed by the VAS scores. This showed a main effect of group (*F* [1, 36] = 6.26, *P* = 0.017, $\eta_{p}^{2}$ = 0.15), with ASMR experiencers reporting more pain than controls *M*_diff_ = 1.24, CI [0.24, 2.25], *P* = 0.017, *d* = 0.73, with no other statistically significant main effects or interaction effects (Fig. 3).

### 3.3. Algometer pain induction

All assumptions have been met therefore, a 2 (group: ASMR experiencers, controls) × 2 (sex: male, female) × 3 (video type: ASMR, control, no video) ANOVA was performed on algometer scores as a measure of pain threshold.

There was a main effect of sex (*F* [1, 36] = 12.83 *P* = 0.001, $\eta_{p}^{2}$ = 0.26), with women having overall lower pain threshold than men (Women: *M* = 8.50, *SD* = 4.24, Men*: M* = 13.13, *SD* = 4.32; *M*_diff_ = −4.66, 95% CI [−7.25, −2.01], *P* = 0.001, *d* = −1.07).

There was a significant main effect of video type (*F* [2, 72] = 7.05, *P* = 0.002, $\eta_{p}^{2}$ = 0.16). Bonferroni-corrected pairwise comparisons revealed significantly lower pain thresholds at baseline (*M* = 9.93, *SD* = 417) compared with ASMR video (*M* = 11.34, *SD* = 5.02) (*M*_diff_ = −1.40, CI [−2.53, −0.28], *P* = 0.010, *d* = −0.32) and compared with the control video (*M* = 11.15, *SD* = 5.32), (*M*_diff_ = −1.24, CI [−2.32, −0.16], *P* = 0.019, *d* = −0.29) and no significant difference between ASMR and control video (*M*_diff_ = 0.16, CI [−0.70, 1.02], *P* = 1.00, *d* = 0.04).

**Figure 4.**
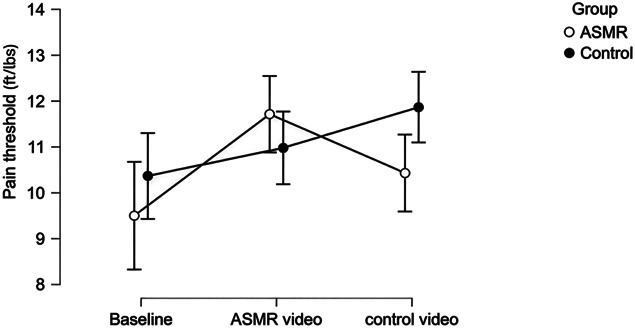
Pain threshold scores across baseline, ASMR video, and control video conditions for ASMR experiencers and controls. ASMR, autonomous sensory meridian response.

The results also showed a significant interaction between group and video type (*F* [2, 72] = 3.99, *P* = 0.023, $\eta_{p}^{2}$ = 0.10). Post hoc comparisons with Bonferroni correction revealed no statistically significant group differences in any of the video conditions; however, it was found that ASMR experiencers showed significantly higher pain threshold in the ASMR video condition (*M* = 11.71, *SD* = 5.02) than at baseline (*M* = 9.50, *SD* = 3.48), *t*(36) = −3.65, *P* = 0.012, *M*_diff_ = −2.32, 95% CI = [−4.313, −0.324], *d* = −0.54. All other comparisons were statistically nonsignificant (Fig. 4).

A second significant interaction between sex and video type was also found (*F* [2, 72] = 6.12, *P* = 0.004, $\eta_{p}^{2}$ = 0.15). Post hoc comparisons with Bonferroni correction revealed statistically significant differences between men (*M =*13.92 *SD* = 4.38) and women (*M =* 8.77*, SD =* 4.29) in the ASMR video condition (*t* [36] = −3.78, *P* = 0.009, *M*_diff_ = −5.28, 95% CI = [−9.672, −0.888 ], *d* = −1.22) and in the control video condition (men: *M* = 14.00, *SD* = 4.69, women: *M* = 8.30, *SD* = 4.36, *t* [36] = −3.86, *P* = 0.007, *M*_diff_ = −5.61, 95% CI = [−10.181, −1.047], *d* = −1.30), while the sex difference in the no video condition (female: *M* = 8.41, *SD* = 4.28, male: *M* = 11.45, *SD* = 3.53, *t* [36] = −2.35 *P* = 0.365, *M*_diff_ = −2.98, 95% CI = [−6.798, 1.007], *d* = −0.69) was nonsignificant.

**Figure 5.**
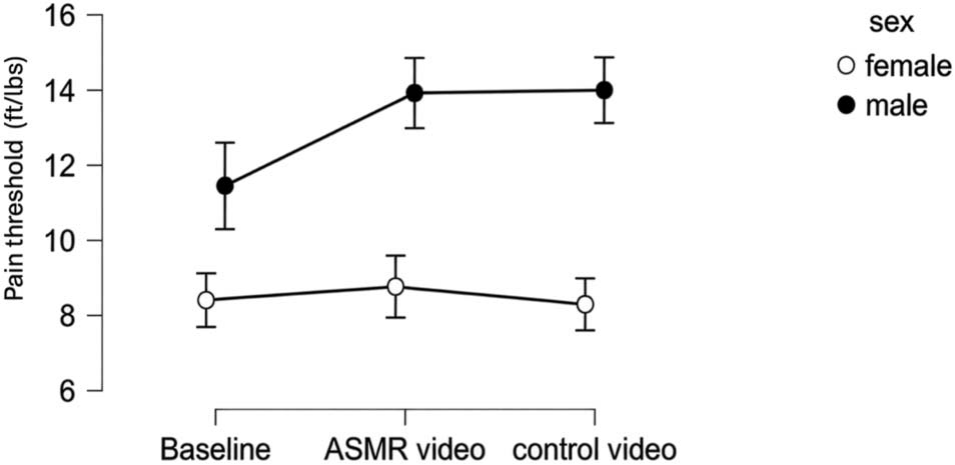
Pain threshold scores across baseline, ASMR video, and control video conditions for men and women. ASMR, autonomous sensory meridian response.

Post hoc comparisons also revealed that men had significantly lower pain thresholds in the no video condition (*M* = 11.45, *SD* = 3.53) compared with the ASMR video condition (*M* = 13.92, *SD* = 4.38) *t*(36) = −4.02, *P* = 0.004, *M*_diff_ = −2.55, 95% CI = [−4.545, −0.556 ], *d* = −0.59, and control video condition (*M* = 14.00*, SD* = 4.69) *t*(36) = −4.21, *P* = 0.002, *M*_diff_ = −2.56, 95% CI = [−4.464, −0.650 ], *d* = −0.59. There was no difference in pain threshold for men between ASMR and control video conditions *t*(36) = −0.02, *P* = 1.000, *M*_diff_ = −0.01, 95% CI = [−1.525, 1.510], *d* = −0.01. Women showed no variation in pain thresholds regardless of condition as all post hoc comparisons for women across conditions were statistically nonsignificant (Fig. 5).

## 4. Discussion

The results presented here suggest 4 key findings. First, analysis of the sex by video-type interaction on the pain threshold data indicates that the statistically significant sex differences were observed in the ASMR video and control video conditions but not at baseline. In addition, men reported significantly lower pain thresholds in the no video condition compared with the ASMR and control video conditions, suggesting that watching the videos significantly increased thresholds for men but not women, who showed no variation in pain thresholds regardless of stimulus, which aligns with previous evidence suggesting that men are more likely to use distraction as a coping mechanism when in pain.^23^ Moreover, in line with existing evidence,^11^ men showed higher pain thresholds than women, although there was no sex difference in VAS pain intensity reporting.

Second, the significant interaction observed between group and video condition for pain threshold data indicates that ASMR experiencers had the highest pain threshold when watching ASMR videos, slightly lower threshold when watching the control video, and the lowest when in the no video condition, although only the difference between ASMR video and no video was statistically significant. Controls showed more consistent pain thresholds across conditions as none of the differences were statistically significant in this group. This aligns with our findings showing that as expected watching an ASMR video induced an appropriate ASMR response in ASMR experiencers, whose tingle frequency was significantly higher in the ASMR video condition than in the control video condition and at baseline. Autonomous sensory meridian response also appears to have served to increase pain thresholds in this group differentially compared with controls or when ASMR experiencers were exposed to other stimulus types, indicating a more unique effect of ASMR videos, as opposed to a general distraction effect caused by visual stimuli, thus aligning with earlier research suggesting that experiencing ASMR may have analgesic properties.^3,21^

Third, while the condition by group by sex interaction was nonsignificant, it does seem that there is potentially sex-based variation in the benefits of watching ASMR and other content for ASMR experiencers. Specifically, while the biggest benefit was observed for both sexes while watching the ASMR video, male ASMR experiencers also saw beneficial consequences in terms of pain thresholds in the control video condition (albeit smaller), while the threshold for female ASMR experiencers was only increased in the ASMR video condition and remained virtually the same and much lower at baseline and in the control video condition. Male ASMR experiencers also showed a much greater increase in their pain thresholds compared with female ASMR experiencers in response to the 2 videos. These findings while statistically nonsignificant highlight the need for a more targeted methodology to examine sex-based differences in ASMR's benefits. Importantly, there were no sex differences overall and male and female ASMR experiencers showed comparable ASMR responses, with similar tingle frequencies across conditions, indicating that the observed sex differences in pain thresholds cannot be attributed to differences in ASMR responding.

Finally, our findings also revealed a significant difference between ASMR experiencers and controls in their reported pain (VAS scores), with ASMR experiencers overall reporting perceptions of higher pain intensity. This is in line both with our own previous findings^21^ and broader research which shows that ASMR is associated with greater self-reported sensitivity to external sensory stimulation^27,31,33^ and increased awareness of bodily sensations.^27^ Current findings are also in line with neuroimaging research showing increased activity within brain regions involved in somatosensation, pain perception, and interoception among ASMR experiencers relative to controls.^25,32,33^ Interestingly, ASMR experiencers have also been recently found to show low self-reported interoceptive accuracy^28^ and greater propensity to become distressed by pain and discomfort,^27^ potentially suggesting a reduced ability to correctly process physiological signals in this group accompanied by interoceptive attention which can be skewed towards maladaptive anxiety-related preoccupation with bodily signals, which may potentially result in greater pain appraisal as we have found in this study. On the other hand, ASMR has also been associated with increased sensation seeking within the tactile domain^28^ and increased reactivity to affective touch,^14^ suggesting a presence of concomitant heightened sensitivity to positive/pleasurable tactile stimulation among ASMR experiencers. These findings also align with research showing increased prevalence of ASMR among synaesthesia^29^ and, in particular, mirror-touch synaesthesia^14^ which in turn has been linked with increased tactile sensitivity^2^ further pointing to a wider ASMR phenotype which encompasses differences in tactile processing, which can lead to both positive and negative outcomes.

Taken together, these findings highlight important implications for research on ASMR, pain, and sex-based variations in responding to both these phenomena. We observed sex-related variation in how ASMR and control videos influenced pain thresholds, consistent with broader evidence of sex differences in pain responding, reporting, and other perceptual elements.^11,23^ Prior work shows that men tend to favour distraction-based coping,^23^ whereas women benefit more from social and emotion-focused strategies,^37^ which may explain why men showed increased pain thresholds even when viewing non-ASMR content, while women benefited only from ASMR videos featuring interpersonal triggers. Although speculative, these patterns suggest that different ASMR triggers may differentially affect men and women. Given the limited research in this area, future work should examine how individual differences in ASMR preferences and responsiveness shape pain and other outcomes, particularly in clinically relevant contexts. It should additionally be noted that reliance on algometry alone limits the generalisability of these findings to other pain induction modalities, and to pain perception more generally, and further investigation should incorporate a broader range of techniques to better investigate these elements.

This study was designed to replicate and build on our own previous work, which had been completed with an underpowered sample due to disruption during the COVID-19 pandemic, which we have successfully achieved. Specifically, we have replicated our previous findings of enhanced pain appraisal among ASMR experiencers using a larger sample size. We have also extended our findings by including sex in our analysis and incorporating pain threshold as a different but related measure of pain. However, considering observed variation in scores which consistently was not found to be significant, it is likely that a more tailored approach may be needed to elucidate sex-based variation in ASMR responding and how it influences pain perception. Furthermore, our results and conclusions indicate that tailoring ASMR content to the participants' preferences may potentially be an important element in ASMR variation, rather than just a methodological opportunity to ensure ASMR responding to the selected stimuli. We did not collect the data needed to complete this kind of analysis, and future research should work to rectify this oversight such that the more granular influence of ASMR effects can be better examined, in particular when considering the potential benefit to clinical samples.

Furthermore, our sample included only clearly identifiable ASMR responders and nonresponders, as individuals who could not be confidently classified were excluded. However, this limits the generalisability of the findings to those with more ambiguous ASMR profiles, such as individuals who do not experience tingling but may still show positive affective responses to ASMR content. It therefore remains to be established how such variability in ASMR responding influences pain processing and related outcomes.

Future research should also examine whether expectancy effects contribute to the elicitation of ASMR and its analgesic outcomes, despite evidence that ASMR experiencers are generally not susceptible to expectancy manipulation.^5,16^ Because we did not assess participants' expectations regarding the ASMR videos, we cannot determine their influence on the present findings. However, recruiting from a general student population likely reduced the probability of prior ASMR-related assumptions. Nonetheless, future studies should explicitly assess expectations to clarify their potential influence.

In conclusion, this study indicates that ASMR stimuli may influence pain responses differently for male and female ASMR experiencers. While controls showed little benefit, ASMR experiencers demonstrated increased pain thresholds when exposed to ASMR stimuli, with the strongest effects observed in men. Although further targeted research is needed, these findings suggest that ASMR could serve as a low-risk, cost-effective pain management strategy for ASMR experiencers. Future work should explore sex differences more fully and examine effects in clinical populations such as chronic pain patients.

## Disclosures

The authors report no conflict of interest. This research meets the guidelines for ethical conduct and report of research. Ethics approval for this study was obtained from the Ethics Reviewers at Bath Spa University. The data is attached to this submission. This study was not registered.

## Supplemental digital content

Supplemental digital content associated with this article can be found online at http://links.lww.com/PR9/A437.
