# Supplementary material for: The modulatory effects of autonomous sensory meridian response (ASMR) on pain
Source: Pain Rep. 2026 Aug 5;11(5):e1487. doi: 10.1097/PR9.0000000000001487 (PMC13446973; doi:10.1097/PR9.0000000000001487)

Table 1

M(SD) for ASMR and control groups, and sex on Tingle Frequency scores

Condition	Sex	Group	Mean	SD
No Video	Female	ASMR-experiencers	1.09	0.30
		Control participants	1.00	0.00
	Male	ASMR-experiencers	1.00	0.00
		Control participants	1.00	0.00
		ASMR-experiencers	1.05	0.22
		Control participants	1.00	0.00
ASMR video	Female	ASMR-experiencers	2.82	1.66
		Control participants	1.33	0.70
	Male	ASMR-experiencers	3.00	1.32
		Control participants	1.09	0.30
		ASMR-experiencers	2.90	1.48
		Control participants	1.20	0.52
Control video	Female	ASMR-experiencers	1.82	1.40
		Control participants	1.22	0.44
	Male	ASMR-experiencers	2.11	0.93
		Control participants	1.27	0.90
		ASMR-experiencers	1.95	1.19
		Control participants	1.25	0.72

Note. N = 20 ASMR-experiencers. N = 20 Control participants. N = 20 Females, N = 20 Males.

Scale range 1 – 7

Table 2

M(SD) for ASMR and control groups, and sex on VAS Scores

Condition	sex	Group	SD	
No Video	Female	ASMR-experiencers	4.74	1.02
		Control participants	2.56	1.45
	Male	ASMR-experiencers	3.99	2.56
		Control participants	3.27	1.88
		ASMR-experiencers	4.40	1.86
		Control participants	2.95	1.70
ASMR video	Female	ASMR-experiencers	4.24	1.47
		Control participants	2.48	1.38
	Male	ASMR-experiencers	3.53	1.92
		Control participants	3.06	1.68
		ASMR-experiencers	3.92	1.67
		Control participants	2.80	1.54
Control video	Female	ASMR-experiencers	4.04	1.63
		Control participants	2.48	1.28
	Male	ASMR-experiencers	3.62	1.88
		Control participants	2.85	1.92
		ASMR-experiencers	3.85	1.71
		Control participants	2.68	1.64

Note. N = 20 ASMR-experiencers. N = 20 Control participants. N = 20 Females, N = 20 Males.

Scale range 0-100

Table 3

M(SD) for ASMR and control groups, and sex on Algometer scores(threshold)

Condition	sex	Group	Mean	SD
No Video	Female	ASMR-experiencers	7.80	3.25
		Control participants	9.16	5.40
	Male	ASMR-experiencers	11.58	2.57
		Control participants	11.35	4.28
		ASMR-experiencers	9.50	3.48
		Control participants	10.37	4.81
ASMR video	Female	ASMR-experiencers	9.05	3.97
		Control participants	8.42	4.88
	Male	ASMR-experiencers	14.96	4.30
		Control participants	13.07	4.46
		ASMR-experiencers	11.71	5.02
		Control participants	10.98	5.11
Control video	Female	ASMR-experiencers	7.28	3.03
		Control participants	9.54	5.51
	Male	ASMR-experiencers	14.28	4.74
		Control participants	13.77	4.86
		ASMR-experiencers	10.43	5.20
		Control participants	11.87	5.47

Note. N = 20 ASMR-experiencers. N = 20 Control participants. N = 20 Females, N = 20 Males

Scale range 0-50

ASMR Verification procedure

The ASMR checklist (Janik McErlean, Ellis & Walsh, 2022; Janik McErlean and Osborne-Ford, 2020; Janik McErlean and Michael, 2018) administered in this experiment included a description of the phenomenon based on which participants were asked to indicate whether they ever experience ASMR, whether it is from watching ASMR videos or in everyday life. Everyone was then asked to indicate whether any of the common listed stimuli trigger tingling sensations in them while viewing ASMR videos, or in daily life. Subsequently everyone was asked to indicate how intense the ASMR experience is on average when engaging with those stimuli on a scale from 0 = No Tingles 1 2 3 = Moderately Intense 4 5 6 = Most Intense ASMR Experience. Participants were also asked to rate how pleasurable ASMR experience is on average using the following scale: very uncomfortable, quite uncomfortable, mildly uncomfortable, neutral, mildly pleasurable, quite pleasurable, and very pleasurable. They were also asked how calming ASMR experience is on average on a scale: not calming at all, somewhat calming, neutral/ not sure, moderately calming, very calming. Finally, at the end of the experiment, everyone was presented with the final question asking them whether they would classify themselves as ASMR-experiencers knowing what it entails and having watched the ASMR video.

Participants classified as ASMR-experiencers responded Definitely Yes and Maybe/I don't know to the initial question related to self-identification as an ASMR-Experiencer while the controls included participants who responded Definitely No and Maybe/I don't know.

ASMR-experiencers also had to either list at least one trigger or select tingling intensity of minimum 1 to at least 1 common trigger. Most participants classified as ASMR-experiencers selected multiple triggers and indicated intensity above 0 for several listed stimuli. Only one participant indicated no triggers but then selected tingle intensities of up to 5 to a number of triggers on the list. Most of the participants classified as controls did not list any ASMR triggers and did not indicate any tingle intensity to the listed triggers, although it is of note that 5 of them did. However, based on their subsequent answers they were classified as controls as they were inconsistent with the phenomenon. Specifically, those classified as ASMR-experiencers had to indicate that the sensation is pleasurable and calming (Swart, Bowling, & Banissy, 2021) and if they selected the 'neutral' option or a negative one they were not included in this group (e.g. they stated that the ASMR experience was very uncomfortable or not calming at all). The difference in the average tingle intensity (calculated across all the listed ASMR triggers)

between the two groups was statistically significant ($t(38) = 7.800$, $p < .001$, ASMR $M = 2.12$, $SD = 0.67$, Controls: $M = 0.40$, $SD = .79$) further demonstrating that the two groups differed in terms of this core ASMR characteristic.

The majority of those classified as ASMR-experiencers also selected 'definitely yes' and most controls selected 'definitely no', to the final question asking about self-identifying as an ASMR-Experiencer having participated in the experiment, although some participants in the two groups answered, 'Not sure'. In hindsight, adding this question was not necessary as the wording of that question may have been confusing resulting in participants giving the less definitive answers. Nevertheless, we still did use this question in the verification process such that no-one classified as ASMR-experiencer indicated 'definitely no' and no-one in the control group indicated 'definitely yes'.

References

Janik McErlean AB, Banissy MJ. Increased misophonia in self-reported autonomous sensory meridian response. PeerJ 2018; 6:e5351. doi: 10.7717/peerj.5351

Janik McErlean AB, Ellis L, Walsh J. "No pain, no gain": the impact of autonomous sensory meridian response on pain perception. Perception 2022; 51 (8): 565-577. doi: 10.1177/03010066221108273

Janik McErlean AB, Osborne-Ford EJ. Increased absorption in autonomous sensory meridian response. PeerJ 2020; 17:8:e8588. doi: 10.7717/peerj.8588

Swart TR, Bowling NC, Banissy MJ. ASMR-Experience Questionnaire (AEQ): A data-driven step towards accurately classifying ASMR responders. British Journal of Psychology 2022;113(1):68-83. doi: 10.1111/bjop.12516

ASMR checklist

Here is a description of a phenomenon called autonomous sensory meridian response (ASMR).

ASMR can be defined as a pleasurable tingling static-like sensation which typically originates on the scalp and can spread down the spine and through the whole body. It is typically induced by certain sounds (e.g. turning pages, crinkly wrapping paper, finger tapping), watching someone perform repetitive mundane actions (e.g. folding towels, going through items in a handbag), watching someone closely inspecting day-to day objects, hearing whisper, watching someone's hair being brushed, having your hair brushed or your back traced with a finger or watching videos with various role plays (visit a doctor, spa or a shop). ASMR is relaxing and calming. ASMR is not frisson (e.g. 'goosebumps' when listening to music).

Having read the description would you say you ever experience ASMR whether it is from watching such videos or in everyday life? Would you classify yourself as somebody who experiences ASMR?

- ○ Definitely Yes
- ○ Not sure
- ○ Definitely No

Please indicate whether or not these common stimuli trigger your ASMR – please select all the items that trigger your tingling sensations while viewing ASMR videos or in your daily life.

- ☐ Crisp, crinkling sounds
- ☐ Personal attention toward you
- ☐ Whispering or soft speaking
- ☐ Tapping sounds
- ☐ Watching someone slowly performing mundane actions
- ☐ Watching someone concentrate on a task
- ☐ Delicate hand movements
- ☐ Physical touch on your body e.g. having your hair brushed, massage
- ☐ Mouth sounds e.g. lip smacking
- ☐ Eating sounds e.g. chewing, swallowing
- ☐ Other (please elaborate)

Please indicate how intense the ASMR experience is on average when engaging with those stimuli.

0 = No Tingles
1 2 3 = Moderately Intense
4 5 6 = Most Intense ASMR Experience.

For example, if whispering triggers the most intense tingles for someone, they would label that stimulus a "6"

Crisp, crinkling sounds 0 1 2 3 4 5 6

Personal attention toward you 0 1 2 3 4 5 6

Whispering or soft speaking 0 1 2 3 4 5 6

Tapping sounds 0 1 2 3 4 5 6

Watching someone slowly performing mundane actions

0 1 2 3 4 5 6

Watching someone concentrate on a task 0 1 2 3 4 5 6

Delicate hand movements 0 1 2 3 4 5 6

Physical touch on your body e.g. having your hair brushed, massage

0 1 2 3 4 5 6

Mouth sounds e.g. lip smacking 0 1 2 3 4 5 6

Eating sounds e.g. chewing, swallowing 0 1 2 3 4 5 6

Other (Tick 0 if you did not indicate any other triggers)

0 1 2 3 4 5 6

On average, how pleasurable is an ASMR experience?

- Very Uncomfortable
- Quite Uncomfortable
- Mildly Uncomfortable
- Neutral
- Mildly pleasurable
- Quite pleasurable
- Very pleasurable

On average, how calming is an ASMR experience for you?

- Not at all calming
- Somewhat calming
- Neutral/Not sure
- Moderately
- Very calming

ASMR is characterised by a tingling sensation which typically starts at the head and can spread throughout the body. It is typically relaxing, calming and pleasurable. Having watched the ASMR video would you classify yourself as somebody who can experience ASMR?

- Definitely Yes
- Not sure
- Definitely No

Algometer administration timeline

Algometer readings were taken at three different points during each condition and were then averaged providing one mean score per condition. The timing of the algometer application was kept constant within each condition and occurred at similar time intervals across the three conditions: at the start, approximately at 1 min and 2 min into the session.

During the no video condition (baseline), the readings were taken at the start and after 1 and 2 minutes.

During the ASMR video which runs from 11:45-14:45 mins in the video, the readings were taken at 11:45 when the video starts which coincides with hair cutting/combing, then at 12:45 when hair washing occurs and the final reading at 13:25 when the shampooing occurs.

During the control video which runs from 2:45- 5:45 the readings are taken at 2:45 as the video begins which coincides with giving instructions, then at 3:55 during the carrot slicing and the final reading is taken at 5:02 when the potato is being scooped.

No video (baseline) algometer application timeline

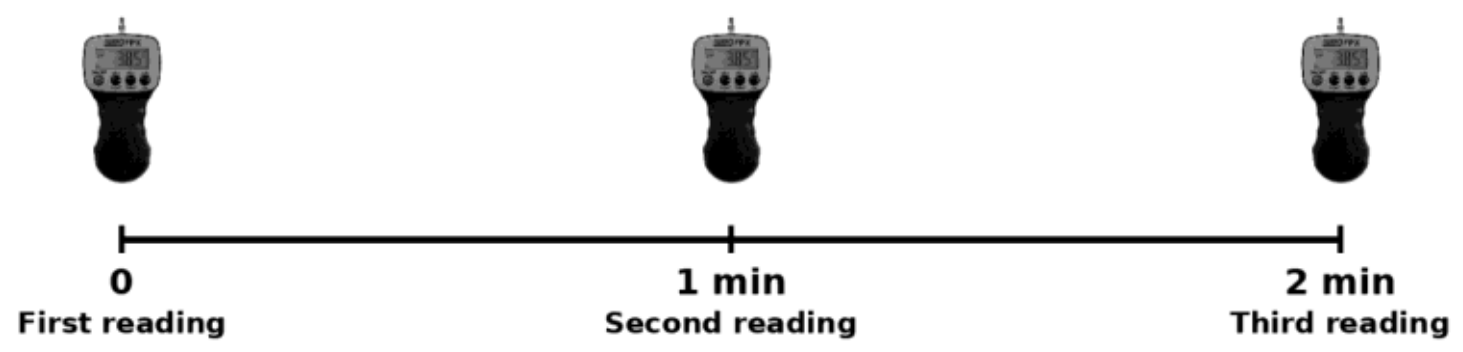

ASMR video algometer application timeline

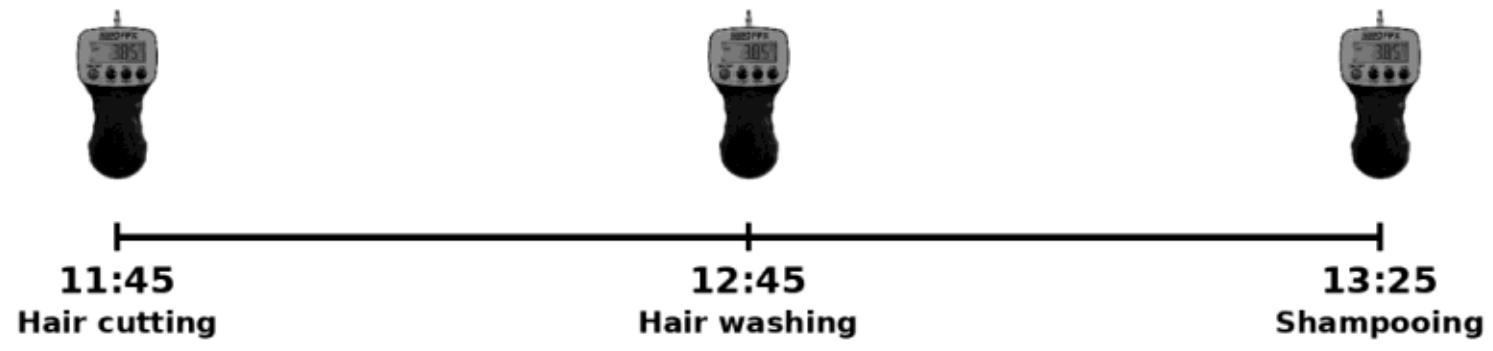

Control video application timeline

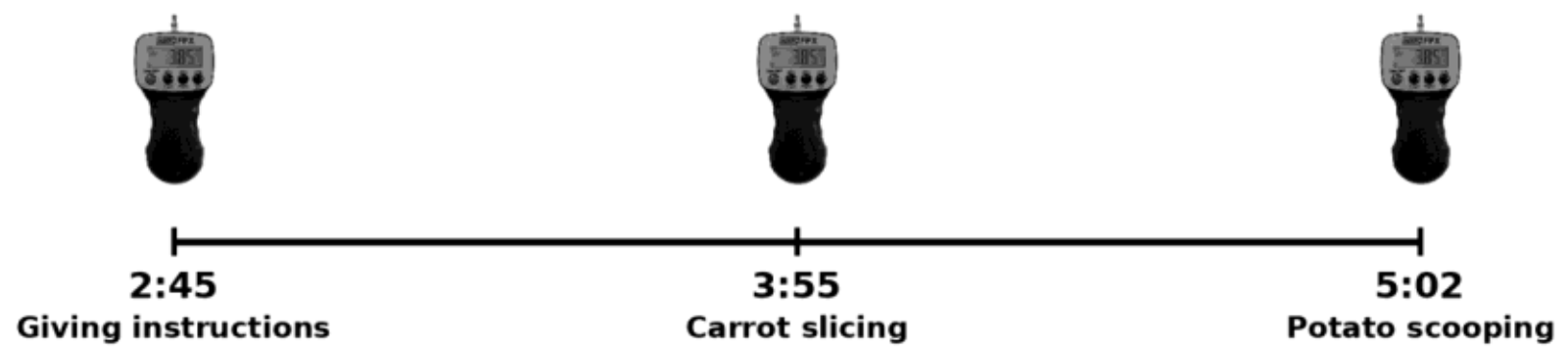

Supplement: Supplementary file 1 [file painreports-11-e1487-s001.pdf]
